# Mammaglobin as a potential molecular target for breast cancer drug delivery

**DOI:** 10.1186/1475-2867-9-8

**Published:** 2009-03-23

**Authors:** Lian Zuo, Ly Li, Qian Wang, Timothy P Fleming, Shaojin You

**Affiliations:** 1 Atlanta Research and Education Foundation, Atlanta VA Medical Center (151), Decatur, GA 30033, USA; 2 Department of Chemistry, University of Ningxia School of Chemistry and Chemical Engineering, Yingchuan, Ningxia, PR China; 3 Department of Chemistry and Biochemistry, University of South Carolina School of Art and Science, Columbia, SC 29208, USA; 4 Department of Surgery, Washington University School of Medicine, St. Louis, MO 63110, USA; 5 Atlanta Research and Education Foundation, Atlanta VA Medical Center (151), Decatur, GA 30033, USA

## Abstract

**Background:**

Mammaglobin (MAM) has been used as a specific molecular marker for breast cancer diagnosis. Recently, several groups of researchers proposed a number of therapeutic strategies targeting this molecule. Some of the strategies are based upon an essential but not demonstrated hypothesis – mammaglobin is associated with the surface of breast cancer cells, which strongly disputes the therapeutic strategies.

**Results:**

We conducted a computer-based predictive analysis and identified a small fragment at the N-end of MAM as a potential transmembrane domain. We provided several evidences to demonstrate the presence of the membrane-associated MAM. We isolated the membrane protein components from known MAM positive breast cancer cells (MDA-MB361 and MDA-MB415). We showed that about 22–64% of MAM proteins, depending upon the types of the cancer cells, directly attached on the membrane of breast cancer cells, by Western blotting assays. To directly visualize the presence of the membrane-bound MAM protein, we incubated the MAM positive cancer cells with FITC labeled anti-MAM antibody, and observed clear fluorescent signals on the surface of the cells. In studying the MAM protein distribution in human breast cancer tissues, we first identified two immunostain patterns that are associated with the membrane-bound MAM: the membrane stain pattern and luminary surface stain pattern. To test whether the membrane-associated MAM can serve as a molecular target for drug delivery, we conjugated anti-MAM antibody to human low-density lipoprotein (LDL) and loaded doxorubicin (Dox) in the core of LDL. Specific binding and cytotoxicity of the MAM targeted and Dox loaded LDL was tested in the MAM positive breast cancer cells *in vitro*.

**Conclusion:**

We first showed that some of MAM protein directly associated with the surface of breast cancer cells. The membrane-associated MAM protein may be utilized as a useful molecular marker for breast cancer targeted drug delivery.

## Background

Watson and Fleming first named the protein encoded by a novel cDNA isolated from a primary human breast cancer as mammaglobin (MAM) [1]. Since MAM protein is homologous to a family of secreted proteins, it is classified as a member of the secretoglobin family. So far, the function of MAM has not been well known. It is assumed that MAM is involved in regulating the host steroid metabolisms and immune functions [2]. Colpitts et al reported that MAM binds to a lipophilin B or BU101 protein in a head to tail format and forms a complex [3,4]. Analyses of the purified complex indicated that the assembly was proceeded with cleavage of the signal peptides from both MAM and lipophilin B proteins. The assembled protein complex formed a small helical globule and create a hydrophobic pocket capable of binding steroid-like molecules and biphenyls [4]. Several years later, Berker et al identified another human uteroglobin-like gene and named it as mammaglobin B (*Mam-B*), which is highly homologous to the *Mam *gene or *Mam-A *characterized by Watson and Fleming [5]. It has been reported that the expression of the *Mam-A *gene is highly restricted to the adult mammary gland [6], whiles the expression of the *Mam-B *gene is found in many organs, such as breast, uterus, salivary gland, lacrimal gland, testis, ovary, and thyroid [5]. More attention, therefore, has been focused on the *Mam or Mam-A *as a diagnostic marker of breast cancer. In a RT-PCR based analysis on the axillary lymph nodes from twenty breast cancer patients, thirteen known metastatic lymph nodes showed *Mam *mRNA positive while all of the remaining pathologic negative nodes were negative for *Mam *[7]. The RT-PCR-based *Mam *mRNA assay was also used for detection of circulating breast cancer cells in the peripheral blood of patients [8].

Recently, MAM has also been investigated as a molecular marker for developing breast cancer targeted therapeutic tools. However, lack of evidence that MAM protein exists on the surface of breast cancer cells strongly disputes on these therapeutic strategies, especially when anti-MAM antibody is used as a targeting ligand for drug delivery. In this study, we demonstrated the presence of the membrane-associated MAM and proved that the membrane-associated MAM can serve as a molecular target for breast cancer targeted drug delivery.

## Methods

### Computer-Based Analysis on Mammaglobin Protein

The protein sequence of mammaglobin was downloaded and reformatted in Fasta sequence and up-loaded to the "HMM-based Protein Structure Prediction" webpage for a SAM-T02 analysis . The secondary structures of MAM protein were predicted and analyzed.

### Cell Culture

Cancer and non-cancerous cell lines were grown at 37°C with or without 5% CO_2_. MDA-MB-361 (MB361), MDA-MB-415 (MB415), T47D, and MDA-MB-231 (MB231) (human breast cancer cell lines, ATCC) were maintained in DMEM:Ham's F-12 medium (50:50; Mediatech) with 10% fetal bovine serum, 2 mM L-glutamine, 100 U/ml penicillin, and 100 μg/ml streptomycin (GIBCO, Life Technologies Inc., Carlsbad, CA). HAEC (human aortic endothelial cell line, Clonetics) and cell culture medium (EGM-2 Bulletkit) were purchased from Cambrex (East Rutherford, NJ). All cell lines were used at early passages (5–10).

### Isolation of the Membrane and Cytosolic Protein Fractions from Cultured Cells

Cells were treated with ice-cold hypotonic lysis buffer (10 mM Tris pH 7.4, 1.5 mM MgCl2, 5 mM KCl, 1 mM DTT, 0.2 mM sodium vanadate, 1 mM PMSF, 1 ug/ml aprotinin, 1 ug/ml leupeptin) for 5 minutes. After drawing the lysate through a 1-mL syring with several rapid strokes, the samples were centrifuged at 2000 g at 4°C for 5 minutes. The supernatant was centrifuged at 100,000 g at 4°C for 90 minutes, and the supernatant was saved as "cytosolic" fraction. The pellets were saved as "membrane" fraction.

### Western Blot Assays

Growth-arrested cells were lysed with 500 μl of ice-cold lysis buffer, pH 7.4 ((in mM) 50 HEPES, 5 EDTA, 50 NaCl), 1% Triton X-100, protease inhibitors (10 μg/ml aprotinin, 1 mM phenylmethylsulfonyl fluoride, 10 μg/ml leupeptin) and phosphatase inhibitors ((in mM) 50 sodium fluoride, 1 sodium orthovanadate, 10 sodium pyrophosphate). Cell lysates (25 μg) were separated using SDS-polyacrylamide gel electrophoresis and transferred to nitrocellulose membranes, blocked overnight in PBS containing 6% nonfat dry milk and 0.1% Tween 20, and incubated for 1 h with primary antibodies. After incubation with secondary antibodies, proteins were detected by ECL chemiluminescence.

### Immunohistochemistry

Immunohistochemical staining for MAM protein was performed as described previously [9,10]. In brief, tissue array sections consisting of 36 human breast cancer and 36 adjacent breast benign tissue cores (Ray Biotech, GA) were deparaffinized and rehydrated. After antigen retrieval and endogenous peroxidase blocking, the sections were blocked with 5% normal horse serum. The slides were incubated with anti-MAM antibody (diluted at 1:500 dilution) at 4°C overnight, then incubated with secondary antibody (ImmPRESS REAGENT kit, VECTOR Lab, CA) and the ImmPACT DAB kit (VECTOR Lab, CA). The immunostained slides were counterstained with hematoxylin and evaluated using a Nikon microscope with an Olympus digital camera.

### Anti-MAM Antibody Incubation and Cell Viability Assay

The cells were cultured in chambered slides and incubated with anti-MAM antibody (Zeta corp. CA, clone 304-1A5) at a concentration of 150 ng/ml for 24 hours at 37°C. The cells were then washed three times with 1× PBS buffer and stained with the Live/Dead Cell Viability/Cytotoxicity Kit according to the instructions from the manufacturer (Molecular Probes Carlsbad, CA). The live cells were shown in green and dead cells were shown in red under fluorescent microscopy. The percentage of dead cells was estimated as follow: Cell viability (%) = (dead cell count/total cell count) × 100.

### Anti-MAM Conjugation to ApoB-100 Protein on the Surface of LDL Particles

In order to conjugate anti-MAM antibody to the apoB-100 protein on LDL particles, a water-soluble carbodiimide was used to activate the carboxyl groups on the surface of apoB-100 protein [11,12]. In brief, 1 mg LDL (Sigma-Alorich, St. Louis, MO) was added to 1 ml of 0.3 M sodium acetate with continuous stirring in an ice-water bath. The acetic anhydride was added in multiple small aliquots (2 μL) over a period of 1 hr with continuous stirring. The reaction mixture was then dialyzed for 24 hr at 4°C against dialyzing buffer. The activated LDL particles were added to 0.4 mg 1-ethyl-3(3-dimethylaminopropyl) carbodiimide (EDC) and 1.1 mg of sulfo-NHS in 1 ml of 0.15 M NaCl. After 60 minute incubation at room temperature, 1.4 μl of 2-mercaptoethanol was added to quench the EDC. Equal mole of anti-MAM was added to the LDL reaction mixture and incubated for 2 hours at room temperature. The reaction was stopped by adding hydroxylamine, excess quenching reagent was removed by dialysis, and the synthesized LDL particles (anti-MAM-LDL) were collected.

### Loading Doxorubincin (Dox) into the Synthetic LDL Particles

Dox was added to 1 ml synthesized LDL particles from a stock solution (0.1 ml, 10 mg/ml) and mixed and incubated in a shaker at 37°C for 4–6 hrs in dark. The mixture was then loaded onto a gel filtration column with G25 Sephadex to separate the unloaded Dox from the Dox loaded synthetic LDL. Fractions of 0.5 ml were collected. The Dox loaded LDL particles (anti-MAM-LDL-Dox) were sterilized by passing through a 0.45 μm acetate millipore filter. The concentration of Dox in the synthetic LDL was then measured as follow: twenty micro liters of the anti-MAM-LDL-Dox particles were added to 780 μl of acidified isopropanol. A standard curve of the Dox concentration in acidified isopropanol versus the absorbance (O.D.) at wavelength 480 nm was obtained. This curve was used to determine the concentration of the synthetic LDL [13]. The morphology and particle size of the synthetic LDL particles were analyzed by electron microscopy using a Philips EM 300 and photographed at 75,000× magnification [14].

### *In Vitro *Testing the MAM Targeted Therapeutic LDL Nanoparticles

The human breast cancer MB415 cells and human aortic endothelial cells HAEC were grown in the 4-well chambered slides and incubated with the MAM targeted therapeutic LDL at concentration 0.3 mg/ml of Dox for 24 hours. The cultured cells were then harvested for cell viability assays. As controls, both cells were also incubated with free Dox at 1 mg/ml, native LDL-DiI at 250 ng/ml, and LDL-Dox at 1 mg/ml.

## Results

### MAM is predicted as a transmembrane protein

**Figure 1:**
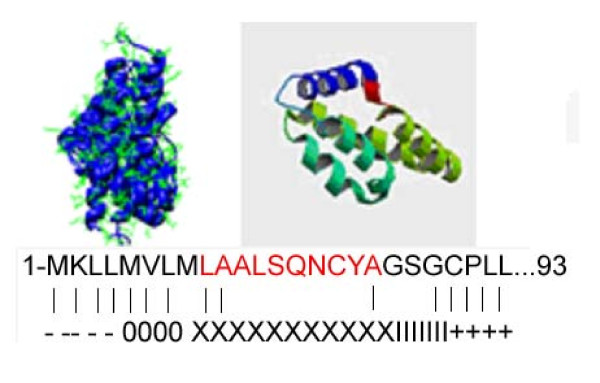
**Computer-assisted structural analysis of MAM protein**. The predicted secondary structure of MAM/lipophilin B dimmer (upper left) and MAM alone (upper right) created by the HMM-based protein structure prediction program, SAM-T02. A helix fragment at the N-end of MAM protein is predicted as a transmembrane domain (the dark blue helix). The protein sequence shown is the N-terminal end of MAM. "+" Inside loop; "-" Outside loop; "O" Outside helix cap; "X" Central transmembrane helix segment; "I" Inside helix cap.

Proteins are usually composed of one or more functional regions, or domains. The identification of domains that occur within proteins can provide insights into their functions. The meta-server technique represents one of the major progresses in the field of protein tertiary structure prediction. To predict the secondary structure of the protein, we conducted a predictive analysis on the MAM protein sequence with the SAM-T02 [15]. Based on the predictive analysis, we identified five helixes or domains on the protein (Figure 1). Among these domains, the fragment at the N-end of MAM protein (the 9–18^th ^amino acid residues) is different from those of the lipophilin and other uteroglobin family proteins (such as pheromaxein C subunit, prostatein C3 subunit, uteroglobin). This fragment mostly consists of the hydrophobic amino acids and is predicted as the trans-membrane helix. So we proposed that MAM proteins are, at least some if not all, associated with cell membrane.

### MAM is associated with membrane in human breast cancer cells

**Figure 2:**
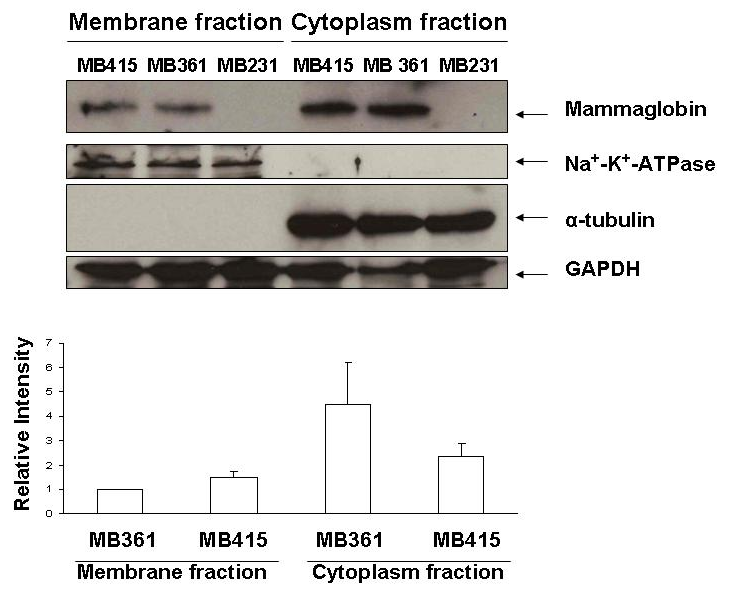
**Detection of membrane associated MAM protein on breast cancer cells**. The Western blot assay shows the specific MAM protein bands (10.5 kDa) in both membranous and cytoplasmic proteins of MDA-MB361 (361) and MDA-MB415 (415) cells; while no specific bands in the membranous and cytoplasmic proteins of MB231 cells. The bar graph represents the quantitative measurements of Western blot assays from four separated experiments (mean ± SE).

To examine whether MAM is a membrane-associated protein in breast cancer cells, we isolated the membrane and cytosol proteins from the cultured MDA-MB415 (MB415), MDA-MB361 (MB361), and MDA-MB231 (MB231) breast cancer cells respectively. We used Na^+^-K^+^-ATPase as the membrane marker and α-tubulin as the cytosol marker. By performing Western blot assays, we detected MAM protein existing at both membrane and cytosol fractions in MB361 and MB415 cancer cells, but not in the MB231 cells (Figure 2). The membrane and cytosol associated MAM proteins were quantitatively evaluated based on the scanned intensities of the specific bands for MAM by normalization of GADPH bands. The membrane-associated MAM protein of MB415 cells was estimated 49.4% more than that of the MB316 cells. The ratios of the membrane-associated MAM protein vs. cytosolic MAM were about 22.2% and 64.1% in the MB361 and MB415 cells respectively.

To test whether MAM proteins are detectable on living breast cancer cells in vitro, we incubated the known MAM positive human breast cancer cells MDA-MB361 (MB361) with the FITC labeled anti-MAM monoclonal antibody, and then monitored the cell fluorescent signals at 1, 4, and 24 hours after the incubation. Obvious fluorescent signals were observed on the MB361 cells as early as 1 hour after the incubation, but the strongest signals were seen after the 24 hours incubation (see Additional file 1).

**Figure 3:**
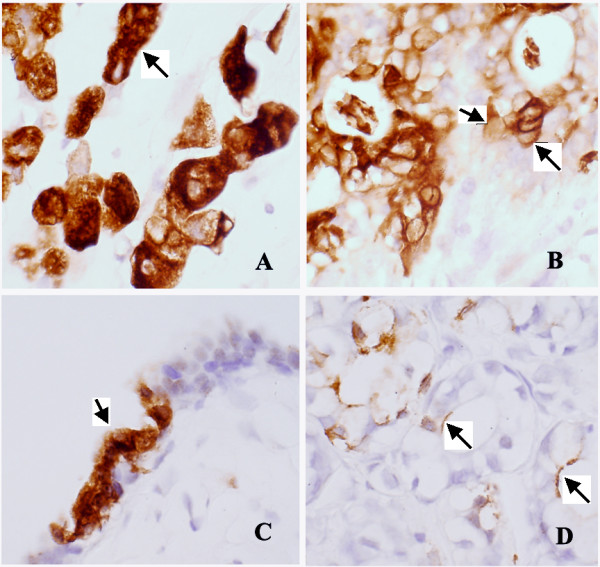
**Immunohistochemical stain of MAM in breast cancer and non-cancerous tissues**. Three types of MAM stain patterns were observed – cytoplasmic (**A and C**), membranous (**B**), and luminary surface stain (**D**). The number of positive cells and intensity of the MAM immunostain in cancer tissues are much higher and stronger than those in the adjacent benign breast tissues. (Images taken by 40× objective lens).

To confirm MAM protein is associated with the membrane *in vivo*, we studied the tissue microarray sections of human breast cancers with anti-MAM antibody by immunohistochemistry. Ten of 36 breast cancer tissue cores were stained positive for anti-MAM antibody while five of 36 breast benign tissue cores were weakly positive. The intensities of the MAM immuno-staining were quite heterogeneous among the breast cancer cores from marginal to abundant positive with overall stronger stain in the breast cancers, as compared with that of the breast benign tissues. There are three types of anti-MAM immuno-staining patterns observed: (1) cytoplasmic stain pattern (Figure 3A and 3C); (2) membrane stain pattern showed at the surface of some breast cancer cells (Figure 3B); and (3) luminary stain pattern showed at the luminary surface of some benign mammary glands (Figure 3D). These data strongly suggest that some of MAM proteins are associated with the cell membrane in both benign and malignant breasts.

### Anti-MAM induced a weak cell apoptotic response in human breast cancer cells

**Figure 4:**
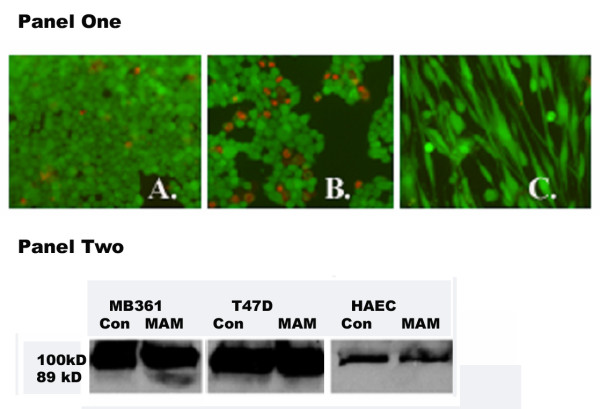
**Panel One: Incubation of MB361 cells with anti-MAM antibody and induction of cell apoptosis**. Cell viability assay was conducted after incubation of MB361 breast cancer cells with anti-MAM. The number of the dead cells (in red color) was found higher in the anti-MAM incubated cancer cells (**B**), as compared with that in the non-incubated MB361 cells (**A**). There were no or few dead HAEC cells (**C**). The images were taken with objective lens 10×. Panel Two: Western blot assay for the pro-PARP-1 (100 kDa) and activated PARP-1 (89 kDa) in the protein lysates of MDA-MB361, T47D, and HAEC cells. The Con – control cells with no anti-MAM antibody inoculation, MAM – cells with anti-MAM antibody inoculation.

After the incubation with 150 ng/ml of anti-MAM antibody, about 30–40% of MB361 cancer cells were detached from the cell culture slides. We assumed the cell apoptosis is induced. To test this assumption, we incubated MB361 cells with anti-MAM antibody. After 24 hour incubation, we conducted cell viability assays and found only about 10% of the attached cells dead (Figure 4 Panel One). In addition, we performed western blot assays to examine the expression of cell apoptosis relevant proteins under this condition from the cultured MB361, T47D, and HAEC cells. PARP-1 is a marker protein for cell apoptosis. The pro-PARP-1 protein is about 100 kDa and the activated form is 89 kDa. The activated PARP-1 was detected only in the MB361 cells incubated with anti-MAM antibody (Figure 4 Panel Two). However, there were no changes in the expression of other apoptosis relevant proteins such as caspase 3 and Bax-1 (data not shown) in the MB361 cells. These data indicated that only limited cell apoptosis was induced with the antibody incubation. The mechanisms that are involved in the cell detachment remain unclear.

### Membrane-associated MAM protein can be targeted for therapeutics in human breast cancer cells

Our primary interest is to examine whether the membrane-associated MAM can be used as a molecular target for drug delivery. So we designed a series of cellular experiments *in vitro*. A MAM-targeted drug carrier was synthesized by conjugating anti-MAM antibody to the surface protein apo-B100 in human LDL particles, of which doxorubicin (Dox) was loaded in the core. As shown by transmission electron microscopy (see Additional file 2), the morphology and size of the LDL particles containing anti-MAM antibody and Dox (anti-MAM-LDL-Dox) remain largely unchanged.

**Figure 5:**
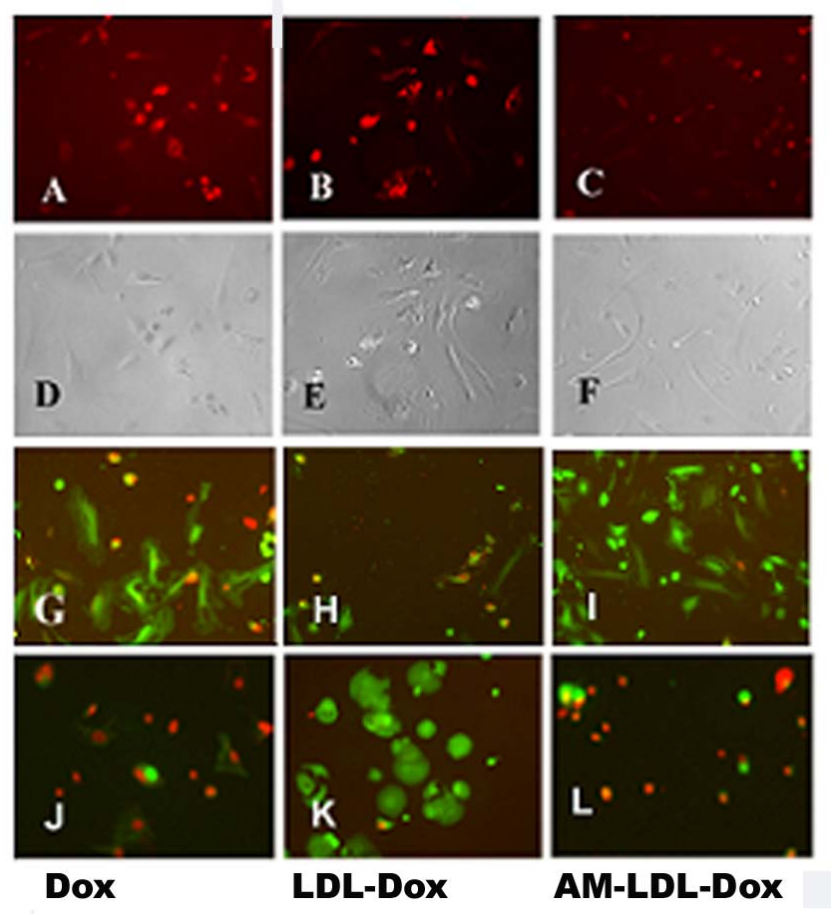
**Specificity and cytotoxicity assay of the synthesized MAM targeted LDL particles**. The images (**A-C**) show the cell binding and endocytosis of free Dox, LDL-Dox, and anti-MAM-LDL-Dox. Obvious Dox uptake was found in HAEC cells when the cells were incubated with free Dox and LDL-Dox (Dox is red fluorogenic), but not in the cells incubated with the anti-MAM-LDL-Dox indicating that conjugation of anti-MAM blocks the binding sites of LDL to LDLR. The LDL-Dox and free Dox incubations are served as positive controls in this experiment. The images (**D-F**) are matched phase images (**A-C**). The images (**G-I**) show the results of the cell viability assays after incubation with free Dox, LDL-Dox, and anti-MAM-LDL-Dox, many dead cells (red color) were found when the HAEC cells were incubated with free Dox and LDL-Dox, but few dead cells were shown when they were incubated with anti-MAM-LDL-Dox (most of the cells are green). The images (**J-L**) show the cytotoxicities of free Dox, LDL-Dox and Anti-MAM-LDL-Dox to MB415 Cells. As shown, most of the cells were dead (small and red) when they were treated by free Dox and anti-MAM-LDL-Dox; while only a few dead cells were found dead when they were treated by LDL-Dox. The images were taken with objective lens 10×.

The specific binding and cytotoxic effect of the anti-MAM-LDL-Dox were tested by using HAEC and MB415 cells. The specific binding of the LDL-Dox to the LDL receptor positive HAEC cells were shown in Figure 5B and the cytotoxic effect of the particles was shown in Figure 5H. The surface modification of LDL with anti-MAM antibody altered the binding capacity of the resultant LDL particles. The anti-MAM-LDL-Dox particles do not bind to the HAEC cells to kill the cells as shown in Figure 5C and Figure 5I respectively. However, the anti-MAM-LDL-Dox particles specifically bound to the MAM positive MB415 breast cancer cells and killed most of the cells in the field (Figure 5L). On the other hand, with the anti-MAM modification on the surface apoB-100 protein, the Dox loaded LDL particles had no/little binding and cytotoxic effects on HAEC cells (Figure 5I), indicating that the antibody medication redirect the binding of LDL particles. Our data strongly suggested that MAM protein on the surface of breast cancer cells may serve as a molecular marker for drug delivery.

## Discussion

Several evidences have shown the secretory nature of MAM protein. For examples, MAM is secreted in the medium of cultured MAM positive breast cancer cells [16]; it is detectable in the serum of breast cancer patients [17]; and the MAM positive stains are largely confined to the cytoplasm of breast cancer cells by the immunohistochemical study [18]. With the predictive analysis on the structure of MAM protein, we proposed that some of MAM proteins stay association with the membrane of breast cancer cells after secretion. In this report, we provided three lines of evidences to support our hypothesis. MAM protein was detected in the membrane fraction of the breast cancer cells at first by Western blot assay; Secondly, the binding of the FITC-labeled anti-MAM antibody was found on the breast cancer cells as visualized by fluorescent microscope; and the third, two MAM immunohistochemical stain patterns were identified in breast tissues (the membrane and luminary stain patterns) that are linked with the membrane-associated MAM proteins. In fact, immunohistochemical stain of MAM protein was studied in human breast cancers previously [18]. The membrane and luminary stain patterns somehow were not described or might be overlooked. Based upon the predictive analysis, we proposed that the N-end of the protein may serve as a potential transmembrane domain. This fragment is, however, partially overlapped with the "signal peptide" predicted by Watson and Fleming [1]. There are two possible pathways for the mammaglobin after being synthesized. The first one may happens after the "signal peptide" is cleaved, then mammaglobin is becoming a cytoplasmic protein and secreted. If the "signal peptide" is not cleaved by some reasons, the second pathway may happen that the protein may be transported and attached to the membrane through this transmembrane domain to become a membrane-associated protein. Our results clearly showed that both cytoplasmic and membrane mammaglobin existed in breast cancer cells, indicating both pathways are functional. The detail molecular mechanisms remain to be demonstrated.

MAM has been investigated as a molecular marker for developing breast cancer therapeutic tools. Viehl et al developed a MAM and Tat fusion protein, which could transduce dendritic cells to stimulate the production of the MAM-specific CD4+ and CD8+ T cells. The simultaneous activation of these T cells may lead to an improved overall immune response to the MAM-positive breast cancer [19]. Goedegebuure et al proposed a novel strategy to kill the targeted breast cancer cells by conjugating anti-MAM antibody to the beta-lactamase gene (βL) [20]. The βL induces cancer killing by converting the prodrug, 7-(4-carboxybutanamido) cephlasporin mustard to a cytotoxic compound [21]. Demonstration of the membrane-associated MAM protein on breast cancer cells strongly supports these therapeutic strategies, particularly when anti-MAM antibody is used as the targeting motif for drug delivery. Herceptin is a FDA approved targeted therapeutic antibody. It binds to Her-2/neu receptor on the membrane of breast cancer cells and causes a rapid cascade of reactions resulting in cell apoptosis [22]. While Herceptin proves to be a therapeutic agent in its own, it also has the ability to serve as a drug carrier for even more effective and less intrusive cancer therapy. However, Her-2 is only expressed in about 20% of breast cancers, which means that the remaining 80% of the cancer patients with Her-2 negative expression cannot take the advantage of this treatment [23]. Mammaglobin may be a complementary biomarker for the targeted breast cancer therapy because of its high and exclusive expression in breast cancer tissues [17].

LDL, with its nanoscale dimension and capacity of penetrating solid tumor [24], has become an attractive nanovector for delivery of a wide range of hydrophobic compounds. As an endogenous carrier for transporting cholesterol and other lipids, LDL circulates in blood, across vascular endothelial linings and into the cells of tissues via LDL receptor-mediated pathways [25]. Because of the high cholesterol demand for synthesizing new cell membrane, some types of cancer cells over express LDL receptor (LDLR) [26]. Therefore, LDL particles have been used as nanovectors for the selective delivery of diagnostic and therapeutic agents to tumor cells that over express LDLR [25,27]. To use LDL as a drug delivery system for treatment of cancers that do not express LDLR, however, LDL has to be modified and redirected to alternative tumor molecular targets. The receptor-binding moieties of apoB-100 protein in LDL have highly basic domains containing Lys residues. If these Lys residues are modified, the binding capacity of this protein to the LDLR is essentially abolished [28,29]. Meanwhile, these basic residues can be used for conjugating other motifs and redirect the modified LDL to alternative tumor specific targets. Zheng et al conjugated folic acid (FA) to the apoB-100 protein of LDL and rerouted the modified LDL from their normal receptors (LDLR) to cancer-associated FA receptor (FR) [28]. The major advantages of LDL as nanovector include that it is completely biodegradable, having no immunogenicity, and containing components to which drugs or diagnostic agents can be attached by physical or chemical manipulation [30]. The drawbacks of LDL, however, may include its limited availability and low drug loading capacity [31]. The cost for *in vivo *animal experiment using LDL such a drug deliver system will be extremely high. In this study, LDL was used as a prototypical vector to test our concept of the MAM-oriented drug delivery.

In our experiments, some MB-361 cells were found detached from the culture dishes with anti-MAM antibody incubation. It was initially considered as the loss of cell adhesion due to cell apoptotic death. To prove this assumption, we incubated the MB361 cells with anti-MAM antibody. By western blot assays we failed to detect any expression level changes of some apoptotic related proteins such as caspase-3 and Bax-1, although a weak band of the activated PARP-1 protein, a marker of cell apoptosis, was detected in the MB361 cell lysate. In addition, the cell viability assays of the cultured cancer cells revealed only limited cell death. These data indicated that the loss of cell adhesion caused by anti-MAM incubation may not due to cell apoptosis.

In summary, we first identified a small fragment at the N-end of MAM as a potential transmembrane domain in a computer based analysis, and then demonstrated the presence of the membrane-associated MAM in both benign and malignant breast epithelium. Although the specific binding of anti-MAM antibody to the membrane-bound MAM *in vitro *didn't trigger apparent cell apoptosis, the synthesized MAM targeting LDL particles seemed to be a functional drug carrier tested *in vitro*. MAM may become an attractive biomarker for development of breast cancer targeted therapies.

## Abbreviations

Anti-MAM: anti-mammaglobin antibody; Anti-MAM-LDL: anti-mammaglobin antibody conjugated LDL particle; Anti-MAM-LDL-Dox: anti-MAM antibody conjugated LDL particle loaded with Doxorubicin; Dox: free doxorubicin; LDL-Dox: LDL particle loaded with Doxorubicin; EDC: 1-ethyl-3(3-dimethylaminopropyl) carbodiimide; HAEC: human aortic endothelial cell line; H&E: hematoxylin and eosin stain; LDL: low-density protein; LDLR: low-density protein receptor; MB361: human breast cancer cell line- MDA-MB-361; MB415: human breast cancer cell line-MDA-MB-415; RES: reticuloendothelial system.

## Competing interests

The authors declare that they have no competing interests.

## Authors' contributions

LZ functioned as a main researcher in this study who works covered most of the in vitro assays; LL functioned as a main researcher in this study whose contribution was mainly focused on the protein conjugation and drug loading; Qian Wang contributed significant amount of time and ideas to this study. He served as a co-investigator in two PI's fundings (the DOD and SCCC funding). Some experiments were performed in his lab. His role in this study was equivalent to that of PI; Timothy Fleming contributed great amount of reagents (such as antibody and cell lines) and provided some advices and ideas to this study; Shaojin You proposed the hypothesis and designed the study. He functioned as the supervisor and PI who provided the main funding support to this study. He performed the immunohistochemical assay and analysis. He also composed the manuscript.

## Supplementary Material

Additional file 1 **FITC Labeled Anti-MAM Antibody and Cell Incubation.** Surface binding assay of the FITC labeled anti-MAM on breast cancer cells. After incubation of the FITC labeled anti-MAM, clear fluorescent signals (the green spotty and patchy dots) were shown on the surface of MB361 and MB415 cells (A and B), but not on the surface of HAEC cells (C). Images (D-F) were the phase images taken from the same culture slides with objective lens 10×. Click here for file

Additional file 2 **Transmission electron microscopy. **Additional figure. Click here for file
