# Supplementary material for: Mammaglobin as a potential molecular target for breast cancer drug delivery
Source: Cancer Cell Int. 2009 Mar 23;9:8. doi: 10.1186/1475-2867-9-8 (PMC2662795; doi:10.1186/1475-2867-9-8)
Supplement: Additional file 1 — FITC Labeled Anti-MAM Antibody and Cell Incubation. Surface binding assay of the FITC labeled anti-MAM on breast cancer cells. After incubation of the FITC labeled anti-MAM, clear fluorescent signals (the green spotty and patchy dots) were shown on the surface of MB361 and MB415 cells (A and B), but not on the surface of HAEC cells (C). Images (D-F) were the phase images taken from the same culture slides with objective lens 10×. [file 1475-2867-9-8-S1.doc]

**Additional file 1**

**Method**

**FITC Labeled Anti-MAM Antibody and Cell Incubation**

Anti-MAM monoclonal antibody was dialyzed against the Reaction Buffer (BioRad protein reagent kit, BioRad, CA) and the FITC solution was added to the antibody at a ratio of 40-80 µg per mg of the antibody and mixed well. The microcentrifuge tube was incubated and rotated at RT for 1 hour (avoid light exposure). The excess FITC was removed via dialysis. Breast cancer and non-cancerous cells cultured in chambered slides were incubated with the FITC labeled anti-MAM antibody at concentration of 150 ng/ml. The fluorescent signals on the living cells were monitored at 1, 4, and 24 hours with a fluorescent microscope and images were taken with a digital camera.


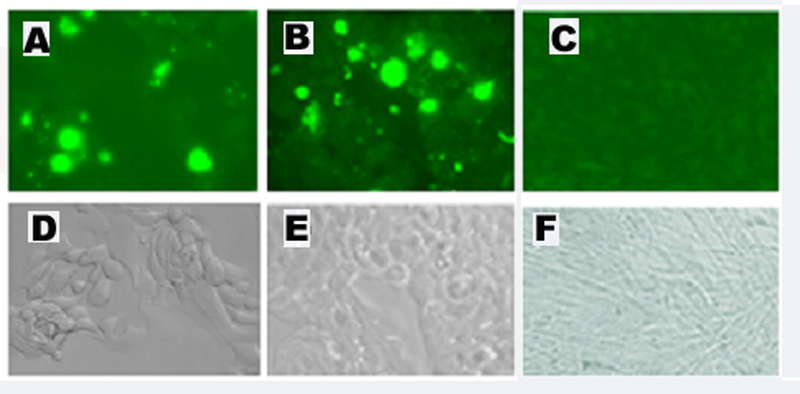


**Figure legend**

**Additional file 1.** Surface binding assay of the FITC labeled anti-MAM on breast cancer cells. After incubation of the FITC labeled anti-MAM, clear fluorescent signals (the green spotty and patchy dots) were shown on the surface of MB361 and MB415 cells (A and B), but not on the surface of HAEC cells (C). Images (D-F) were the phase images taken from the same culture slides with objective lens 10x.
